# Pseudotumor Sarcoidosis: The Contribution of Thoracic MRI to the Presumption of Benignity of a Pulmonary Lesion Process

**DOI:** 10.7759/cureus.37485

**Published:** 2023-04-12

**Authors:** Btissame Es-sabbahi, Maha Ezzine, Bouchra Aamara, Badreddine Alami, Mounia Serraj

**Affiliations:** 1 Pulmonology Department, Hassan II University Hospital, Fez, MAR; 2 Radiology Department, Hassan II University Hospital, Fez, MAR

**Keywords:** diffusion-weighted mri, pulmonary mri, condensation, cannonballs, pseudotumor sarcoidosis

## Abstract

Sarcoidosis is a benign systemic disease; its diagnosis is based on a suggestive radiological presentation, and the isolation of an epithelioid and gigantocellular granuloma (EGGC) without caseous necrosis with the elimination of other causes of granuloma. However, sometimes the radiological presentation is atypical and misleading, posing problems in terms of differential diagnosis. In this report, we present a case of pseudotumor sarcoidosis, in which MRI played an essential role in characterizing the lesion and suggesting its benignity. We also discuss the role of MRI in evaluating atypical forms of sarcoidosis.

## Introduction

Sarcoidosis is a benign, systemic disease of unknown cause characterized by epithelioid and gigantocellular granuloma (EGGC) infiltrates without caseous necrosis [1]. While it can affect the whole body, mediastino-pulmonary involvement is the most frequent one [2]. In most cases, the radiological appearance is characteristic and often suggestive. However, atypical and misleading radiological presentations are not exceptional [2], in particular a tumoral form leading to confusion with a malignant etiology [3]. The role of pulmonary MRI in these cases is not established. We report a clinical case that proves that pulmonary MRI can play a major role in the demonstration of the benign character of these lesions, thus making it possible to rule out the principal differential diagnosis with regard to certain aspects in terms of the tumoral origin, which is associated with the presentation of cannonballs in secondary form, or primitive in the case of the single lesion.

## Case presentation

**Figure 1:**
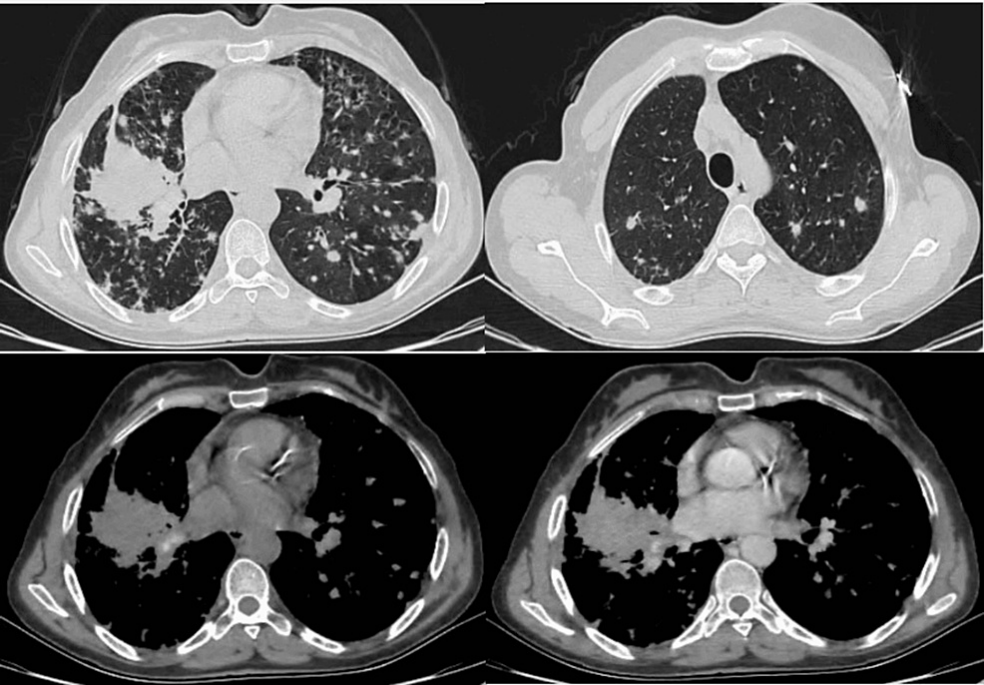
Chest CT axial section The images show a spiculated right upper lobar lesion process, with multiple scattered nodules in both thoracic hemifields CT: computed tomography

The patient was Mrs. F, a 56-year-old female who consulted for a chronic dry cough complicated by exertional dyspnea evolving for one year without extra-respiratory signs; she had no comorbidities or any risk factors, being neither a smoker nor professionally exposed. Her general condition was found to be well. The thoracic CT scan (Figure 1) revealed a large area of pulmonary opacification, with irregular borders, blurred margins and no air bronchogram, proximal parahilar on the right side, associated with multiple bilateral nodules and micronodules forming a "galaxy sign", and mediastinal lymphadenopathy; however, there was no beaded septum. This raised the suspicion of a malignant lung tumor with secondary parenchymal and mediastinal lymph node localization. A bronchial fibroscopy with biopsies was performed, but these came back non-contributory. The decision was made to proceed with a scan-guided biopsy. A thoracic MRI was proposed during the multidisciplinary consultation meeting because of the ability of this examination to orientate the site of the biopsy as well as to study the cellular character of the lesion by the diffusion method, which would allow us to predict whether the lesion was benign or malignant.

**Figure 2:**
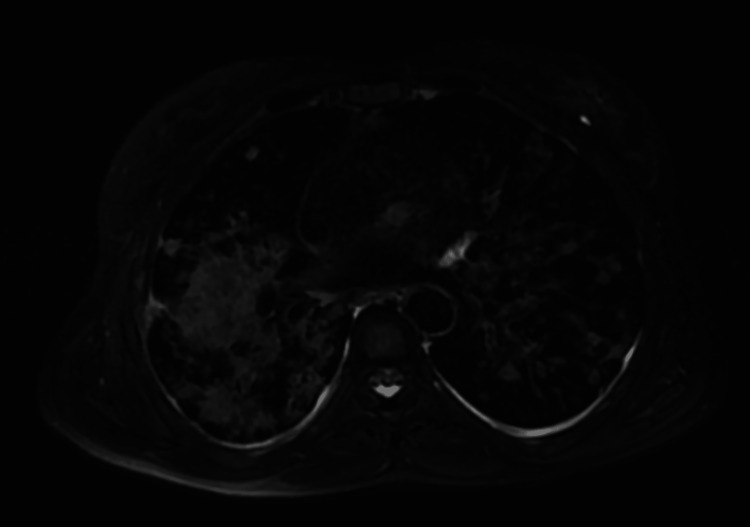
Thoracic MRI: T2 FS sequence The image shows pulmonary parenchymal condensation of intermediate signal, with individualization of several scattered nodules in the two pulmonary hemifields MRI: magnetic resonance imaging

**Figure 3:**
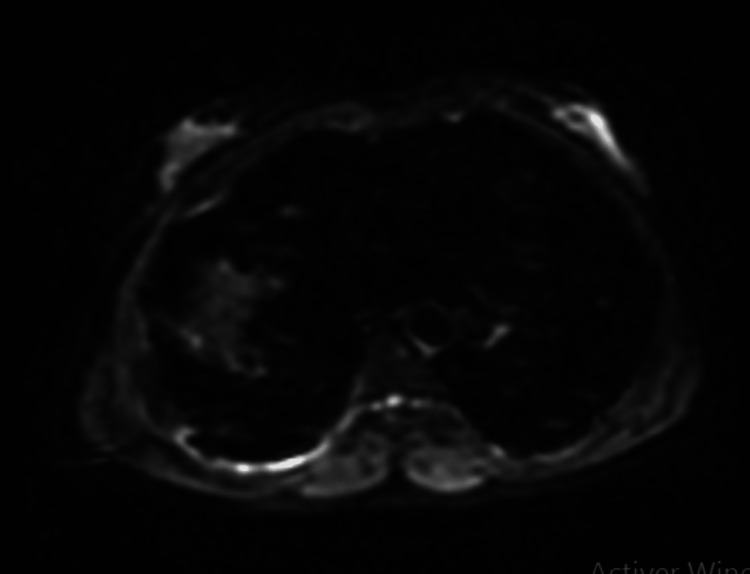
Thoracic MRI: diffusion sequence The lesion does not show diffusion restriction MRI: magnetic resonance imaging

The MRI showed a right upper lobar tissue mass with multiple nodules without diffusion restriction (Figures 2, 3); this lesion had a high apparent diffusion coefficient (ADC) value at 1.8 mm^2^/s, reflecting its hypocellular character and thereby indicating a benign lesion. A scan-guided biopsy was performed, which confirmed the benign nature of the process; it was ultimately found to be a non-necrotizing granulomatous lesion mimicking sarcoidosis in its tumoral form. The patient underwent a sarcoidosis workup to look for other localizations, as well as a functional impact assessment.

## Discussion

Sarcoidosis is a systemic disease of unknown cause, characterized by infiltration of affected organs by epithelioid and gigantocellular immune granulomas (also called tuberculoid granulomas) without caseous necrosis. It is epidemiologically heterogeneous and its clinical presentation is variable. It can affect the whole body, but mediastino-pulmonary involvement is by far the most frequent, present in about 90% of patients, and isolated in half of the cases [1]. It affects women in the third and fourth decades of life and is more prevalent in populations of Caribbean or African origin, in whom the disease is more likely to be chronic [1]. The diagnosis requires the elimination of known causes of granuloma.

Radiological abnormalities are most often suggestive, and sometimes the only manifestations of the disease discovered by chance on a chest X-ray. This allows the disease to be classified into four stages. CT is more sensitive than the standard X-ray in terms of characterizing the observed abnormalities. It can better describe the elementary lesions as well as their anatomical distribution. Contrast injection is useful to analyze adenopathies. The CT findings can be extremely variable, ranging from typical aspects because of their frequency and the lymphatic distribution of the lesions to atypical aspects that are less frequent and pose problems of differential diagnosis.

The most frequently found lesions are diffuse micronodules of lymphatic distribution predominantly in the middle and upper parts of the lungs, as well as adenopathies that are bilaterally symmetrical and non-compressive [2]. In some cases, the nodules may coalesce and give rise to pulmonary nodules and masses, sometimes multiple, and pose problems in differential diagnosis, mainly with regard to neoplastic pathology. The forms with a balloon-like appearance are the most frequent, but unilateral and single attacks have also been described [3]. In our case, the CT scan showed a right upper lobar process, with spiculated contours, with multiple disseminated nodules, associated with mediastinal lymphadenopathy, and a metastatic neoplastic pathology was strongly suspected. However, all around the lesion, there were nodules surrounded by a ring of satellite micronodules forming the galaxy sign, which made us consider the diagnosis of sarcoidosis and perform an MRI in order to study the cellular character or the lack of it by diffusion method, as the ADC value is inversely proportional to cellularity. The latter showed hypocellularity, which supported the theory of the benign nature of the lesion. A scan-guided biopsy was performed, which confirmed the diagnosis of sarcoidosis.

Diffusion MRI is a technique that can measure molecular diffusion in vivo. It allows calculating at each point of the image the distribution of the diffusion directions of water molecules. The movement of these water molecules depends on both the integrity of the cell and the cell density. ADC is a parameter that refers to the specific diffusion capacity of a biological tissue, which has been shown to be useful in differentiating a benign mass from a malignant one. ADC is inversely proportional to cellularity; tumors generally have a higher cell density. However, no consensus has been reached regarding the optimal threshold value of ADC between benign and malignant lesions. In a recent meta-analysis, Shen et al. [4] reported that malignant lung lesions had significantly lower ADC values compared to benign lesions [1.21 mm2/s (95% CI: 1.19 to 1.22) vs. 1.76 mm2/s (95% CI: 1.72 to 1.80); p<0.05]. in our case, the ADC value was high, which supported the benign character of the lesion.

The place of MRI in the diagnosis of pulmonary sarcoidosis is not clear at the moment and remains to be defined. According to the case illustrated above, diffusion MRI can be of great help with misleading forms of pulmonary sarcoidosis, especially in pseudotumor forms, by showing the benign character of its lesions, which allows us to rule out the main differential diagnosis in this case, which is related to the tumor origin. The evolution of these lesions is most often towards resolution either spontaneously or with treatment.

The spontaneous evolution of pseudotumor forms of sarcoidosis is unpredictable. In some series, the evolution was spontaneously favorable with a radiological clearance time ranging from one to two years [5]. In Battesti et al.'s study of 746 patients with sarcoidosis, 22 of 33 patients with pseudotumor sarcoidosis were followed up. The definitive disappearance of lesions was noted in 15 patients treated for two years with corticosteroids. In six untreated patients, the evolution was spontaneously favorable with a time of improvement of three to nine months. Only one patient, for whom corticosteroid therapy was contraindicated, developed pulmonary fibrosis [6]. Nodular forms can cavitate, and cavities can be colonized by fungi, such as aspergilloma.

## Conclusions

The diagnosis of pseudotumor pulmonary sarcoidosis is often difficult. Discordance between the discreteness of the clinical presentation and the extent of the radiological lesions should, however, help with the diagnosis. Histological evidence is necessary to rule out other etiologies, especially tumors. Thoracic MRI with diffusion is a non-invasive and non-irradiating diagnostic tool that can predict the benignity of these tumor lesions. However, the definitive role of this examination remains to be defined in this context.
